# Flexible Antibacterial Coatings

**DOI:** 10.3390/molecules22050813

**Published:** 2017-05-16

**Authors:** Jindřich Musil

**Affiliations:** Department of Physics and NTIS Centre of Excellence, Faculty of Applied Sciences, University of West Bohemia, Univerzitní 22, CZ-306 14 Plzeň, Czech Republic; musil@kfy.zcu.cz

**Keywords:** antibacterial coatings, efficiency of bacteria killing, physical properties, mechanical properties, resistance to cracking, magnetron sputtering

## Abstract

This article reviews the present state of the art in the field of flexible antibacterial coatings which efficiently kill bacteria on their surfaces. Coatings are formed using a reactive magnetron sputtering. The effect of the elemental composition and structure of the coating on its antibacterial and mechanical properties is explained. The properties of Cr–Cu–O, Al–Cu–N, and Zr–Cu–N antibacterial coatings are used as examples and described in detail. The efficiency of killing of bacteria was tested for the *Escherichia coli* bacterium. The principle of the formation of thick, flexible antibacterial coatings which are resistant to cracking under bending is explained. It is shown that magnetron sputtering enables production of robust, several-micrometer thick, flexible antibacterial coatings for long-term use. The antibacterial coatings produced by magnetron sputtering present huge potential for many applications.

## 1. Introduction

In recent years, a great effort has been devoted to the development of antibacterial coatings which efficiently kill bacteria on their surfaces. Main attention has been concentrated on the development of coatings with the highest possible killing activity in the shortest times and without the need for an inducing radiation. Mainly, coatings containing silver and copper have been investigated in detail [1,2,3,4,5,6,7,8,9,10,11,12,13,14,15,16,17,18,19,20,21,22,23,24,25,26,27,28,29,30,31,32,33]. However, no attention has been devoted to mechanical properties of antibacterial coatings which decide their lifetime. For antibacterial coatings, a long lifetime is a key requirement in many practical applications when these coatings are deposited on contact surfaces of rigid or flexible substrates and must simultaneously exhibit two functions: antibacterial and protective. Recently, it has been shown that flexible antibacterial coatings can provide good mechanical protection of substrates [34,35,36,37,38]. The formation of antibacterial coatings with a high efficiency of killing of bacteria that simultaneously demonstrates good mechanical properties is, however, quite a difficult task [13,24,39,40,41]. High efficiency of bacteria killing requires high contents of Ag or Cu in the coating and this fact almost always results in a strong reduction of its hardness and thus of its protection ability [29]. When such coatings are deposited on flexible substrates, they easily crack and/or delaminate from protective surfaces. Therefore, it is vitally important to develop flexible antibacterial coatings resistant to cracking.

Flexible antibacterial coatings represent a new generation of protective coatings. In this article, the principle of the formation of flexible, protective, and antibacterial coatings is explained in detail. It is shown that the key to forming a flexible antibacterial coating is the optimization of (i) its elemental composition and (ii) the energy delivered to the coating during its growth. The mechanical properties of the coating, e.g., the hardness (*H*), the effective Young’s modulus (E*), the elastic recovery (*W_e_*), and the H/E* ratio, which ensure its enhanced resistance to cracking, i.e., its flexibility, were found [34,35,36]. These new properties were demonstrated on reactively sputtered several-micrometer-thick flexible, antibacterial and protective Al–Cu–N [37] and Zr–Cu–N [38] coatings in a dual-magnetron system with a closed magnetic field [42]. It is shown that the Al–Cu–N and Zr–Cu–N coatings with enhanced resistance to cracking very effectively kill the *Escherichia coli* bacteria in the daylight as well as in the dark. The 100% efficiency of killing of the *E. coli* bacteria is reached already after 3 h of bacteria contact with the surface of the Al–Cu–N coating containing 9.6 at.% Cu [37]. The 2–3 μm thick flexible, antibacterial, and protective coatings with a long lifetime represent a huge potential for many applications. At present, these coatings could be industrially deposited on contact surfaces of both rigid and flexible substrates. For instance in hospitals; transport means such as airplanes, buses, trains, and trams; cash and ticket machines; furniture in restaurants, theatres, schools; and other objects, and could efficiently prevent bacteria transfer from their surfaces to humans.

## 2. Antibacterial Cr–Cu–O Oxide Coatings

The Cr–Cu–O coatings were reactively sputtered in a dual-magnetron system in an Ar + O_2_ sputtering gas mixture. Both magnetrons were equipped with the same Cr/Cu targets (Ø 50 mm) each composed of a Cr (99.9 at.%) circular plate fixed to the cathode body with a Cu (99.9 at.%) fixing ring with inner diameter Ø_i Cu_. The concentration of Cu in the Cr–Cu–O coating was controlled by the inner diameter Ø_i Cu_ of the Cu fixing ring and the partial pressure of nitrogen, p_O2_. The dual magnetron was powered by a floating DC pulsed power supply RMP-10 (Huttinger Electronics, Inc., Freiburg, Germany) operated in a bipolar mode at a repetition frequency *fr* = 1/*T* = 20 kHz (*T* = 50 μs); here *T* is the period of pulses [29].

### 2.1. Antibacterial Activity of Cr–Cu–O Coatings

**Figure 1:**
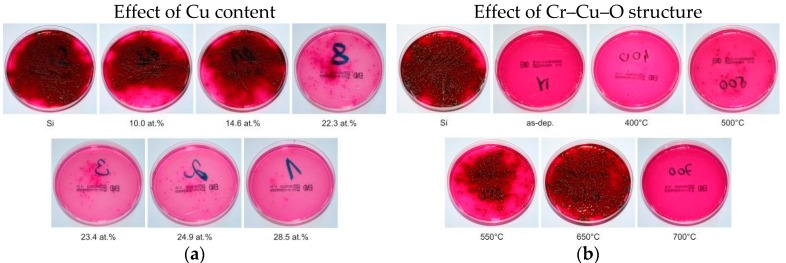
Photos of Petri dishes with evolution of colony-forming units (CFUs) of the *Escherichia coli* bacteria cultivated on Endo agar covered with bacterial suspension, which was in contact with (**a**) the pure Si substrate and the as-deposited Cr–Cu–O coating with increasing Cu content on Si substrate and (**b**) the thermally annealed Cr–Cu–O coating with 20 at.% Cu as a function of the annealing temperature, in the dark for 5 h. Reprinted from [29], Copyright 2013, with permission from Elsevier.

**Figure 2:**
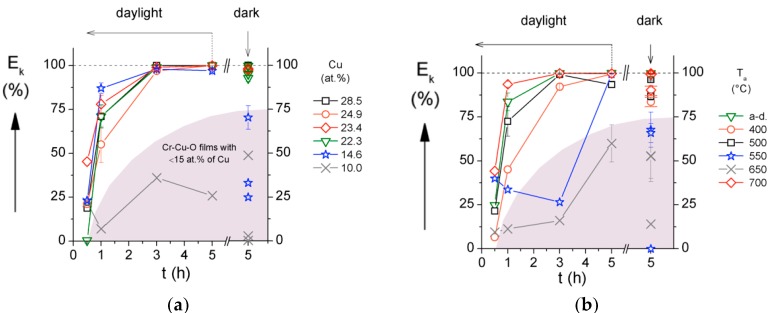
Antibacterial efficiency E_k_ of the *E. coli* bacteria killing of (**a**) the as-deposited Cr–Cu–O coatings with various Cu contents (**b**) the Cr–Cu–O coatings with 20 at.% Cu and various structures induced by post-deposition rapid thermal annealing (RTA) to different temperature T_a_ as a function of the contact time t in the daylight and in the dark. Reprinted from [29], Copyright 2013, with permission from Elsevier.

The antibacterial activity of a coating strongly depends on its elemental composition and structure, see, for instance, reference [29]. These facts are demonstrated by dependences of the killing of *E. coli* bacteria on the surface of the Cr–Cu–O coating, see Figure 1. Generally, it is accepted to characterize the antibacterial function by the number of the so-called colony-forming units (CFUs) at the tested coating surface. For every antibacterial coating, the time necessary to kill the bacteria on its surface is also very important. This time strongly depends on the Cu content in the coating as well, see Figure 2.

From these figures it is seen that:

1. The number of CFUs of the *E. coli* bacteria (black dots) decreases with increasing Cu content in the coating and at the surface of the Cr–Cu–O coatings with >15 at.% content of Cu practically all bacteria are killed; here CFU is the colony forming unit.
2. Figure 1 shows that (i) no *E. coli* bacteria are killed on the surface of a pure Si (the surface of Si is fully covered with black dots, i.e., the CFU of *E. coli* bacteria); (ii) the complete killing of the *E. coli* bacteria is achieved on the surface of the as-deposited coatings with a high (>15 at.%) Cu content (Figure 1a) and the coatings with 20 at.% Cu content thermally annealed up to ~400 °C (Figure 1b); (iii) the efficiency of bacteria killing (E_k_) gradually deteriorates with increasing of the annealing temperature (T_a_) and is the lowest at T_a_ ≈ 650 °C and (iv) the full killing of bacteria is again achieved on the surface of the Cr–Cu–O coating thermally annealed at 700 °C. These changes in the efficiency E_k_ of *E. coli* bacteria killing are caused by the variation of the coating structure with annealing temperature T_a_ (Figure 1b and Figure 2b). The change of E_k_ with increasing T_a_ is due to the change of the coating structure from X-ray amorphous at T_a_ < 550 °C to the following crystalline phases at T_a_ ≥ 550 °C. The coatings annealed at T_a_ ranging from 550 °C to 650 °C are multi-phase coatings composed of a mixture of two crystalline CuO and CuCr_2_O_4_ oxide phases with admixture of the delafossite CuCrO_2_ phase at T_a_ = 650 °C. The CuO and CuCr_2_O_4_ phases disappear at T_a_ = 700 °C when a single-phase Cr–Cu–O coating with the pure delafossite CuCrO_2_ phase is formed. The evolution of the structure of the Cr–Cu–O coating with increasing T_a_ is given in [29].
3. The efficiency E_k_ of the *E. coli* bacteria killing strongly depends on the time of contact of the bacteria with the surface of antibacterial coating. The efficiency Ek increases from 0 to more than 50% during 1 h and reaches ~100% after ~3 h contact of the *E. coli* bacteria with the surface of the Cr–Cu–O coating with high (≥15 at.%) Cu content (Figure 2a).
4. The efficiency E_k_ of *E. coli* bacteria killing on the surface of the Cr–Cu–O coating with ≥ 15 at.% Cu content is the same both in the daylight and in the dark at the contact time t = 5 h (Figure 2a,b). It means that no special irradiation, not even daylight irradiation, is necessary to kill *E. coli* bacteria settled on the surface of the Cr–Cu–O coating.

### 2.2. Mechanical Properties of Cr–Cu–O Coatings

The mechanical properties of a coating are its hardness, *H*; the effective Young’s modulus E* = E/(1 − *ν*^2^) and the elastic recovery *W_e_*; here *E* is the Young’s modulus and *ν* is the Poisson’s ratio. The mechanical behavior of the coating is characterized by the elastic recovery *W_e_*, the ratio H/E* which is proportional to the strain to failure [43,44], and the ratio *H*^3^/E*^2^ which is proportional to the resistance of the material to plastic deformation [45]. The plastic deformation is reduced in highly elastic materials with high hardness *H* and low effective Young’s modulus E* [34]. It means that a low modulus E* is very desirable as it allows the given load to be distributed over a wider area and to increase the resistance of coating against cracking.

**Figure 3:**
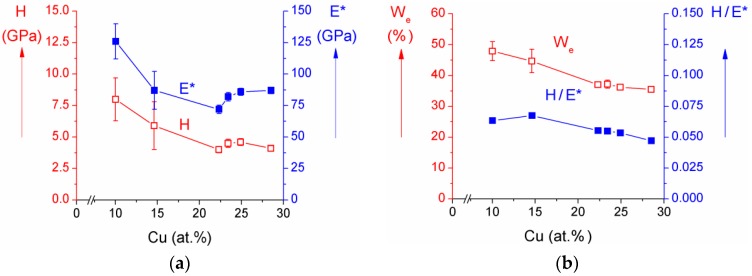
(**a**) Hardness (*H*), effective Young’s modulus (E*) and (**b**) elastic recovery (*W_e_*) and H/E* ratio of as-deposited Cr–Cu–O films as a function of Cu content. Reprinted from [29], Copyright 2013, with permission from Elsevier.

The hardness *H*, effective Young’s modulus E* and elastic recovery *W_e_* of the as-deposited Cr–Cu–O coatings with different Cu content are displayed in Figure 3a,b. From these figures is seen that (i) the hardness *H* and the effective Young’s modulus E* decrease with increasing Cu content up to ~22 at.% Cu, slightly increase in the narrow interval between 22 and 25 at.% Cu and remain almost constant in the interval between 25 and 30 at.% Cu; (ii) the elastic recovery *W_e_* is low and decreases from 48% to 36% with increasing Cu content from ~10 at.% to ~30 at.% Cu; and (iii) the ratio H/E* of the Cr–Cu–O coatings in the whole range of added Cu is very low ≤0.07. It means that all Cr–Cu–O coatings exhibit a low resistance to cracking due to low ratio H/E* < 0.1) and low elastic recovery *W_e_* < 50%.

This experiment clearly shows that the antibacterial Cr–Cu–O coatings with ≥20 at.% Cu are quite hard (~4.5 GPa) and thus can avoid being destroyed under external loading such as fretting. However, these coatings exhibit a low resistance to cracking and therefore crack very easily. This is a great drawback of oxide antibacterial coatings and the reason why it is urgently needed to develop flexible antibacterial coatings with enhanced resistance to cracking and thereby widen their application potential.

## 3. Flexible Antibacterial Coatings

The flexible antibacterial coatings represent a new generation of the antibacterial coatings. These coatings simultaneously fulfill three functions: (1) the antibacterial function, i.e., high efficiency of bacteria killing; (2) the flexible function, i.e., high flexibility of coating; and (3) the protection function against fretting (wear), i.e., several-micrometer thick and robust coatings with enhanced hardness *H* and wear resistance, and long lifetime of operation have been developed. The antibacterial function is controlled by Cu content in the coating and its structure. The flexible function is controlled by (i) the low value of the effective Young’s modulus E* ensuring that the coating exhibits high ratio H/E* ≥ 0.1 and high elastic recovery *W_e_* ≥ 60%, (ii) the microstructure of coating which must be dense and void-free, and (iii) the macrostress (*σ*) generated in the coating during its growth. Therefore, the formation of such coatings is a very difficult but possible task. To form flexible antibacterial coatings the hardness *H*, H/E* ratio, elastic recovery *W_e_* and Cu content in the coatings must be optimized and the elemental composition of the antibacterial Me–Cu–X correctly selected; here Me = Cr, Ti, etc. and X = O, N, etc. One very effective way which makes it possible to form the flexible antibacterial coatings is to replace oxygen in the coating with nitrogen and to form nitride coatings instead of oxide coatings. More details are given in [37,38]. Some important properties of flexible antibacterial Al–Cu–N and Zr–Cu–N are briefly given below.

In summary, it can be concluded that the flexible antibacterial coatings must exhibit simultaneously the following properties [34,35,36,37,38]: (1) sufficiently high Cu content to kill efficiently bacteria on its surface; (2) low E*, H/E* ≥ 0.1 and *W_e_* ≥ 60%, dense, void-free microstructure, and compressive macrostress [46]; and (3) high hardness *H* of about 15 GPa or greater. Antibacterial coatings based on nitrides, for instance, the Al–Cu–N [37] and Zr–Cu–N [38] coatings exhibit such properties. The substitution of O with N results in an enhanced resistance of the Zr–Cu–N nitride coating to cracking due to a strong increase of *H*, *W_e_*, and ratio H/E*, and also in a strong reduction of Cu content in the coating needed for 100% killing of the *E. coli* bacteria on its surface.

## 4. Flexible Antibacterial Al–Cu–N and Zr–Cu–N Coatings

The Al–Cu–N and Zr–Cu–N coatings were reactively sputtered in a dual-magnetron system in Ar + N_2_ sputtering gas mixture. Both magnetrons were equipped with the same Me/Cu targets (Ø 50 mm) composed of Me (99.9 at.%) circular plates fixed to the cathode body with a Cu (99.9 at.%) fixing ring with inner diameter Øi Cu. The concentration of Cu in the Me–Cu–N coating was controlled by the inner diameter Øi Cu of Cu fixing ring and the partial pressure of nitrogen p_N2_. The dual magnetron was powered by a floating DC pulsed power supply RMP-10 (Huttinger Electronics, Inc.) operated in a bipolar mode at (i) the repetition frequency *fr* = 1/*T* = 20 kHz (*T* = 50 μs) in sputtering of the Al–Cu–N coating and (ii) the repetition frequency *fr* = 1/*T* = 40 kHz (*T* = 25 μs) in sputtering of the Zr–Cu–N coating; here DC is the direct current and *T* is the period of pulses. More details on the formation of the antibacterial Zr–Cu–N coatings are given in [38].

### 4.1. Antibacterial Function

**Figure 4:**
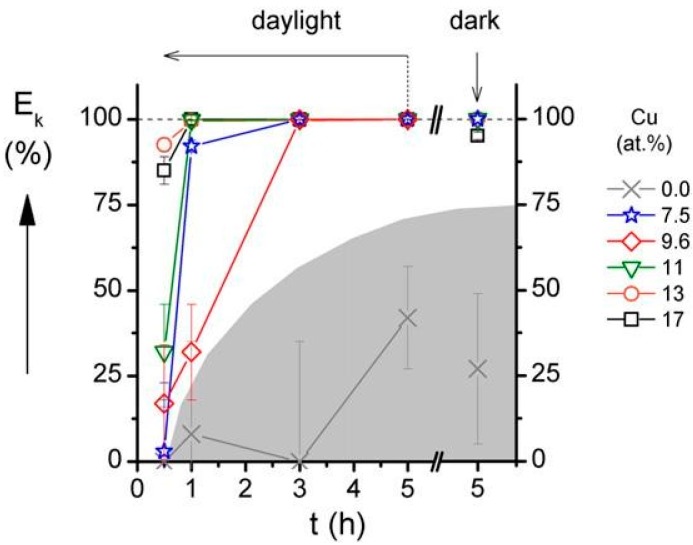
Antibacterial efficiency (E_k_) of the AlN and Al–Cu–N coatings with various Cu contents sputtered on Si (100) substrate as a function of contact time (*t*) with their surfaces. Reprinted from [37], Copyright 2015, with permission from Elsevier.

**Figure 5:**
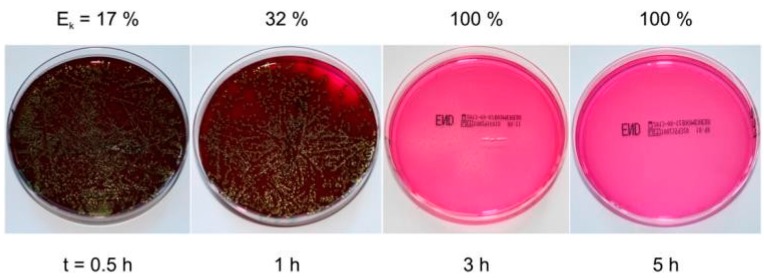
Photos of Petri dishes with the *E. coli* bacteria cultivated on Endo agar covered with bacterial suspension which was in contact with the surface of an Al–Cu–N coating containing 9.6 at.% Cu for different contact times. Reprinted from [37], Copyright 2015, with permission from Elsevier.

The antibacterial activity of the surface of the Al–Cu–N coating was investigated as a function of Cu content in the coating and as a function of contact time during which the *E. coli* bacteria were settled on the surface of Al–Cu–N coating. Results of these experiments are displayed in Figure 4 and Figure 5. The main results of these experiments are the following:

1. The efficiency (E_k_) of the *E. coli* bacteria killing on the surface of Al–Cu–N coating increases with increasing Cu content similarly as was found for the Cr–Cu–O coatings. However, there is a substantial difference in the minimum Cu content necessary to kill all bacteria: ~10 at.% Cu for the Al–Cu–N nitride coatings and ~20 at.% Cu for the Cr–Cu–O oxide coatings. It is a result of the replacement of oxygen O with nitrogen N in the antibacterial Me–Cu–X coating.
2. The time necessary to kill all *E. coli* bacteria on the surface of Al–Cu–N coating with 9.6 at.% Cu is about three hours. However, this time is strongly reduced as much as down to one hour when the Cu content in the Al–Cu–N coating is greater than 10 at.% Cu and does not reduce any further when the Cu content is increased. This finding is very important for an industrial design of antibacterial coatings.
3. The efficiency E_k_ of the bacteria killing is the same in the day light and in the dark. It was confirmed by the measurement of E_k_ in the dark at contact time *t* = 5 h, see Figure 4.
4. Similar results were obtained also for the antibacterial Zr–Cu–N coatings.

### 4.2. Mechanical Properties

**Figure 6:**
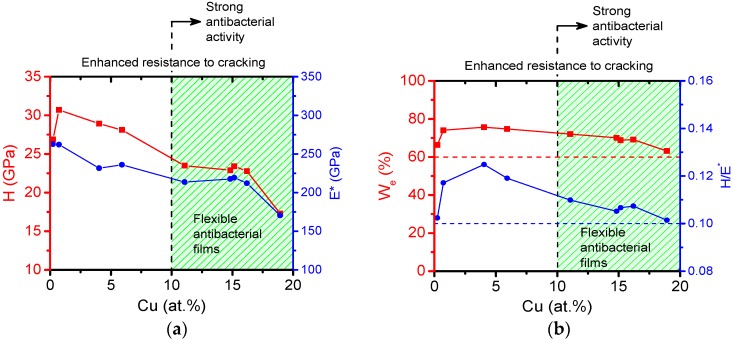
(**a**) Hardness *H*, effective Young’s modulus E*; and (**b**) elastic recovery *W_e_* and H/E* ratio of Zr–Cu–N coatings sputtered on Si (100) substrates as a function of Cu content. Reprinted from [38], Copyright 2015, with permission from AIP Publishing LLC.

The mechanical properties of antibacterial Me–Cu–N coatings are illustrated on mechanical properties of the Zr–Cu–N coatings, see Figure 6. In this figure (a) the hardness *H*, effective Young’s modulus E* and (b) the elastic recovery *W_e_* and the H/E* ratio are displayed as a function of Cu content in the Zr–Cu–N coating. To see the interrelationship between the mechanical and antibacterial properties of Zr–Cu–N coatings, the region of Cu content in which the coatings exhibit a strong antibacterial activity (100% killing of all *E. coli* bacteria) is marked by dashed lines and in green.

The three main observations which follow from results displayed in Figure 6 are the following:

1. The hardness *H* and the effective Young’s modulus E* decrease with increasing Cu content from ~30 GPa to ~17 GPa and from ~260 to ~170 GPa, respectively. Despite this fact, the Zr–Cu–N coatings exhibit high ratio H/E* ≥ 0.1, high elastic recovery *W_e_* ≥ 60%, and also compressive macrostress (σ < 0) as shown in [38]; thereby they also show an enhanced resistance to cracking for all Cu contents ranging from 0 to 19 at.% Cu. Besides, the hardness H of the Zr–Cu–N coatings ranging from ~25 to ~17 GPa is quite high and it makes possible to prevent the coating from being removed from the surface of a substrate by fretting (wear).
2. The efficiency E_k_ of killing of *E. coli* bacteria, however, strongly depends on the Cu content. Only the Zr–Cu–N coatings with Cu content ≥10 at.% kill bacteria very efficiently.
3. Only the Zr–Cu–N coatings with ≥10 at.% Cu are three-functional-flexible/antibacterial/hard coatings. Similar results were obtained also for the flexible antibacterial hard Al–Cu–N nitride coatings [38].

### 4.3. Resistance to Cracking

**Figure 7:**
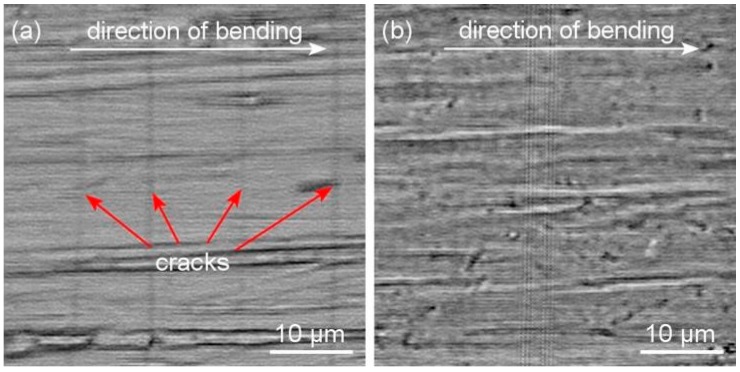
Optical microscope (OM) images of the surface morphology of the Zr–Cu–N coating with (**a**) a low resistance to cracking (H/E* = 0.082 and *W_e_* = 56%) and (**b**) an enhanced resistance to cracking (H/E* = 0.112 and *W_e_* = 70%) sputtered on Mo strip after bending around a fixed cylinder of radius r_fc_ = 10 mm. Reprinted from [38], Copyright 2015, with permission from AIP Publishing LLC.

**Figure 8:**
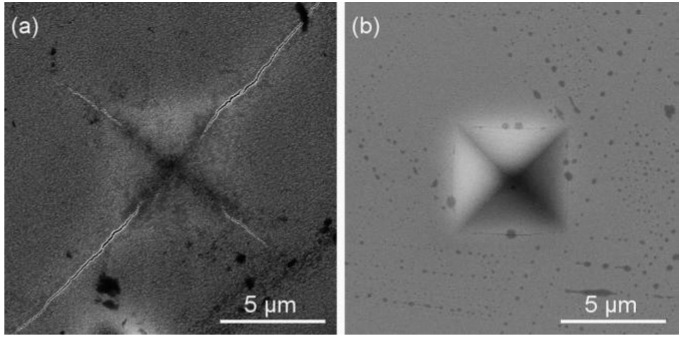
Scanning electron microscopy (SEM) images of the surface morphology of the Zr–Cu–N coating with (**a**) a low resistance to cracking (H/E* = 0.082 and *W_e_* = 56%) and (**b**) an enhanced resistance to cracking (H/E* = 0.112 and *W_e_* = 70%) sputtered on Si (100) substrate after indentation by the diamond indenter at high load L = 1 N. Reprinted from [38], Copyright 2015, with permission from AIP Publishing LLC.5. Comparison of Antibacterial Cr–Cu–O and Zr–Cu–N Coatings

The resistance of sputtered Me–Cu–N coatings against cracking was assessed by the bending and indentation tests [35]. For the bending test, the Me–Cu–N coatings were sputtered on a thin Mo strip (50 × 10 × 0.15 mm^3^) and the coated strip was bent around a fixed cylinder with radius r_fc_. Two Zr–Cu–N coatings with different mechanical properties, i.e., (i) low ratio H/E* = 0.082 and low elastic recovery *W_e_* = 56% and (ii) the high ratio H/E* = 0.112 and high elastic recovery (*W_e_* = 70%), were compared, see Figure 7. The same results were obtained when the resistance to cracking of the same Zr–Cu–N coatings was assessed by the indentation test, see Figure 8. In the indentation test, the coatings were loaded by the diamond indenter at a high load L = 1 N perpendicularly to the coating surface; more details are given in [35]. The cracks in the Zr–Cu–N coating with the low ratio H/E* = 0.082 and the low elastic recovery *W_e_* = 56% are clearly seen.

In this section, basic properties of the Cr–Cu–O oxide and the Al–Cu–N nitride antibacterial coatings are briefly summarized. Two typical antibacterial oxide and nitride coatings were compared: (i) the Cr–Cu–O coating with 19.5 at.% Cu and (ii) the Al–Cu–N coating with 9.6 at.% Cu. The mechanical properties of both coatings strongly differ, see Table 1. Main differences are the following:

1. The Cr–Cu–O coating exhibits low hardness *H* = 3.2 GPa, low elastic recovery *W_e_* = 36%, low ratio H/E* = 0.046, and almost zero macrostress *σ* ≈ 0, which is typical for coatings with a columnar microstructure and a low resistance to cracking in bending and brutal mechanical loading.
2. The Al–Cu–N coating exhibits high hardness *H* = 21.9 GPa, high elastic recovery *W_e_* = 74%, high ratio H/E* = 0.122, and a compressive macrostress *σ* = −1.7 GPa, which is typical for coatings with a dense, void-free microstructure and enhanced resistance to cracking.
3. The antibacterial Cr–Cu–O coating is brittle. On the other hand, the antibacterial Al–Cu–N coating is flexible.

**Figure 9:**
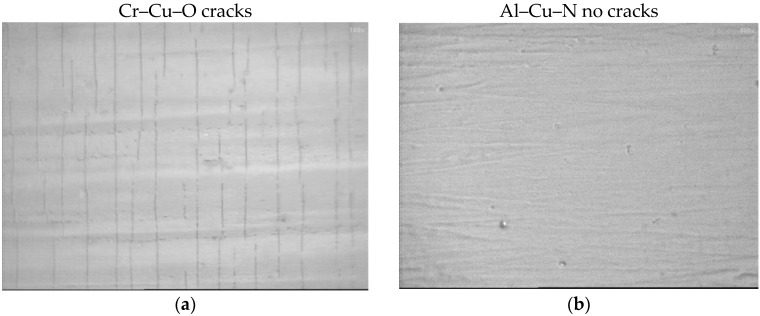
SEM images of the surface morphology of (**a**) the Cr–Cu–O coating with low H/E* ratio (H/E* = 0.046) and low elastic recovery (*W_e_* = 36%), and (**b**) the Al–Cu–N coating with high H/E* ratio (H/E* = 0.122) and high elastic recovery (*W_e_* = 74%) deposited on a thin Mo strip (55 × 15 × 0.15 mm^3^) after bending around cylinder of radius r_fc_ = 10 mm.

These conclusions were fully confirmed in bending of both coatings deposited on thin Mo strip, see Figure 9. In this test, the coated Mo strip was bent around a cylinder of radius r_fc_ = 10 mm. Clear cracks perpendicular to the bending direction are created in the Cr–Cu–O coating which demonstrate that this coating is brittle. On the other hand, no cracks are formed in the flexible Al–Cu–N coating, see Figure 9.

## 5. Conclusions

In summary, it can be concluded that robust, several-micrometer thick, antibacterial/protective/flexible three-functional nitride coatings with enhanced resistance to cracking and a long lifetime can be prepared using magnetron sputtering. These coatings can be used for the protection of many contact surfaces to prevent transfer of bacteria from these surfaces to healthy people. The antibacterial/protective/flexible three-functional nitride coatings can be deposited on both rigid and flexible substrates. These films represent a huge potential for many applications, for instance in hospitals; transport means such as airplanes, buses, trains, and trams; cash and ticket machines; furniture in restaurants, theatres, schools; and other objects. The magnetron technology, which is now well mastered, can be immediately implemented for the industrial production of these coatings. It can be expected that, in the near future, new flexible/antibacterial/protective/three-functional coatings, which will exhibit the best compatibility with materials of the objects whose surface should efficiently kill bacteria settled on it, will be developed.

## Figures and Tables

**Table 1:** Mechanical properties of ~2500 nm thick antibacterial Cr–Cu–O and Al–Cu–N coatings with 100% killing of *E. coli* bacteria sputtered at the substrate temperatures T_s_ = 500 °C and 400 °C, respectively, and assessment of their resistance to cracking by bending around a cylinder of radius r_fc_ = 10 mm.

Coating	Cu (at.%)	*H* (GPa)	E* (GPa)	*W_e_* (%)	H/E*	σ	Cracks in Bending
Cr–Cu–O	19.5	3.2	70	36	0.046	0.1	yes
Al–Cu–N	9.6	21.9	180	74	0.122	−1.7	no
